# Risk factors differ for viable and low viable crushed piglets in free farrowing pens

**DOI:** 10.3389/fvets.2023.1172446

**Published:** 2023-04-21

**Authors:** Cornelia Spörri-Vontobel, Michael Simmler, Beat Wechsler, Madeleine F. Scriba

**Affiliations:** ^1^Centre for Proper Housing of Ruminants and Pigs, Federal Food Safety and Veterinary Office, Ettenhausen, Switzerland; ^2^Digital Production Group, Agroscope, Ettenhausen, Switzerland

**Keywords:** piglet loss, pre-weaning mortality, crushing, primary crushing loss, secondary crushing loss, underweight, weakness, survival analysis

## Abstract

Newborn piglets have a high risk of being crushed by the sow, and this risk implies welfare and economic consequences. The aim of this study was to investigate the importance of differentiating between low viable (secondary crushing losses) and viable crushed (primary crushing losses) piglets for the evaluation of risk factors for crushing related to characteristics of the sow, the litter, and the environment. Eleven Swiss farmers recorded sows’ production data (parity class, gestation length, numbers of live-born and stillborn piglets), data (age, sex, weight, cause of death, and signs of weakness) for every live-born piglet that died in the first week after birth (piglet loss), and ambient temperature. Piglet losses were assigned to five categorical events: *piglet loss*, subdivided into *not crushed* and *crushed*, the latter being further subdivided into *low viable crushed* and *viable crushed*. Piglets recorded by the farmer as *crushed* were assigned to the events *low viable crushed* and *viable crushed* based on the piglet’s body weight and signs of weakness (diseases, malformations). Data of 9,543 live-born piglets from 740 litters were eventually used to statistically model the hazard of dying at any given time in the first week after birth due to one of these events (mixed-effects Cox model). Five potential risk factors were analyzed as co-variates: parity class, gestation length, number of live-born piglets, number of stillborn piglets, and daily number of hours with ambient temperature >30°C. We identified two risk factors for dying from the event *viable crushed* that were not identified as risk factors for *low viable crushed*, namely shorter gestation length and higher daily number of hours with ambient temperature  > 30°C. *Vice-versa*, we identified additional live-born piglets in the litter as risk factor for *low viable crushed*, but not for *viable crushed*. Our results show the importance of differentiating between low viable and viable crushed piglets for the interpretation of risk factors for crushing losses. Therefore, we suggest that for breeding purposes and in research, this differentiation should be made.

## 1. Introduction

For economic and welfare reasons, one of the main goals in pig production is to decrease pre-weaning mortality (PWM) of piglets (1–4). The principal cause of death in the period from birth until weaning is crushing by sows, as consistently described in the scientific literature and reviewed by Muns et al. (2). It accounts for around 50% of all piglet deaths, usually happening in the first week after birth (1, 5, 6). Crushing is described as the final act in a complex chain of interactions between the piglets, the sow, and the environment (2, 7). However, several studies reported that not more than between 18 and 70% of the crushed piglets were healthy and potentially viable (1, 7–9). These findings suggest that a considerable percentage (30–82%) of piglets that were crushed were predisposed to being crushed because of weakness (7). Consequently, the mechanical damage due to crushing is only in a part of the cases the exclusive cause of death (10).

Hypothermia, starvation, and diseases are factors that weaken the piglet (2, 10), leading directly or indirectly to death. The weaker a piglet, the less capable it is to react to posture changes of the sow and to avoid being crushed or trampled (8, 10–16). To protect piglets from the risk of being crushed, farrowing crates are used almost everywhere in the world (17–19). Multiple studies showed that pre-weaning mortality is higher in non-crated than in crated housing systems of the farrowing and lactating sow (reviewed by 17, 18, 20). However, some studies reported that the overall survival rate of piglets was not higher in crated systems than in the tested non-crated systems (12, 13, 21). As shown in two studies (12, 21), piglets have a higher risk of being crushed but a lower risk of dying from causes other than crushing in non-crated systems. Although piglets of weak constitution might be crushed in pens without farrowing crates, they are likely to die from other weakness-related causes of death in crated pens (12, 13).

To reduce crushing losses and PWM in general, the causes of piglets’ death need to be studied in detail (4, 5, 22). The differentiation of the crushed piglets into healthy and weak individuals is thereby of importance, because risk factors for crushing may vary for small, underweight piglets compared to viable, well-fed ones (8). Crushing is considered the primary cause of death for a crushed, viable and healthy piglet of normal weight (8). In contrast, crushing is considered to be the secondary cause of death for a crushed piglet with signs of weakness or low viability such as underweight, malformations, or diseases (3, 8, 21, 23, 24). To date, primary and secondary crushing losses were differentiated in only a few studies (e.g., 3, 8, 21, 23, 24).

Examples of risk factors for crushing are environmental factors such as season and temperature and sow factors such as parity class (2). Studies found contradictory results regarding the effects of these environmental and maternal factors on piglet survival. For example, Weber et al. (13) found more crushing losses in summer than in the other seasons in Switzerland, while Rangstrup-Christensen et al. (8) observed the lowest percentage in summer in Denmark. Additionally, Rangstrup-Christensen et al. (8) detected a higher risk for crushing in multiparous than in primiparous sows. However, Pandolfi et al. (25) found that piglets were less likely to die with signs of crushing in later parities than in the first or second one. Besides differences in the study design, the environmental conditions, and the genetics of the sows, the lack of differentiation between primary and secondary crushing losses might explain these discrepancies.

In addition to ambient temperature and parity class, a large litter size is frequently discussed as a risk factor for general PWM (26–28) and for crushing losses (27, 29). Moreover, a short gestation length (30) and a high number of stillborn piglets were found to be associated with a higher PWM risk (21, 31, 32). The five risk factors addressed so far (ambient temperature, parity class, gestation length, number of live-born piglets, and number of stillborn piglets) are suitable for a study based on farmers’ records. They require little interpretation by the farmer and, therefore, are potentially highly accurate (23).

The aim of this study was to investigate the relevance of a differentiation between low viable and viable crushed piglets for the evaluation of risk factors for crushing losses related to characteristics of the sow, the litter, and the environment. We hypothesized that there are differences in risk factors between the events labelled as *viable crushed*, i.e., being crushed in viable state, and *low viable crushed*, i.e., being crushed in low viable state.

Additionally, we expected that risk factors for dying from other causes than crushing (*not crushed*), typically related to weakness, would be more similar to those for *low viable crushed* than to those for *viable crushed*.

## 2. Materials and methods

### 2.1. Setting of the study

The study is based on data provided by 11 Swiss farmers who collected data on piglet mortality in the first week after birth (0–7 days after birth) by using a detailed protocol. They participated voluntarily in the study and received no financial compensation. Data collection started between May 2018 and July 2019, lasted 5–6 months, and ended after the majority (75–100%) of the producing sows on the farm were recorded at least once, or the end of the study period was reached (December 2019). The farms had an average herd size of 84.5 producing sows (range: 20–168). Small (<50 sows; n = 3), medium (50–100 sows; *n* = 4), and large (>100 sows; *n* = 4) herds, as defined for Swiss conditions, were evenly represented. Most farms (*n* = 8) used F1 crosses between Swiss Large White (SLW) and Swiss Landrace (SL) as damline and pure breed SLW (*n* = 10) as sireline. Three farms used pure breed SLW sows or Duroc boars, and some farms used more than one damline (*n* = 1) or sireline (*n* = 3). One exception was a farm on which a large share of pure SL pigs was used. Over the whole lactation period, sows were kept in free farrowing and lactating pens with a total area of at least 5.5 m^2^, as required by the Swiss Animal Protection Ordinance (33). Mean pen size on the study farms was 7.2 m^2^ (± 0.31). Different types of free farrowing pens were used on the different farms and in some cases within the same farm. Nine farms used pens with no option for temporary crating, whereas four farms used simple pens and five farms used FAT2 pens (34) with a separation between dunging and nesting area. Two farms used pens allowing temporary crating, but on one farm this option was never used and on the other farm it was used in exceptional cases only (leg weakness or aggression of the sow against her piglets).

### 2.2. Structure of the protocol

The farmers were given written instructions on how to record data on the protocol sheets. This included photographs to illustrate terms and definitions. Farmers were instructed to record dead piglets with fully intact slippers (eponychium) on claws as stillborn (23, 35–37). Most farmers were already familiar with similar protocols used for breeding and production data. Each protocol contained specific information for a sow and a given litter. The sow was identified by the ear tag number and the number of the farrowing room. The upper part of the protocol asked for information on the sow’s parity class, the anticipated and actual farrowing date, the number of live-born and stillborn piglets, the number of cross-fostered piglets, and the number of piglets alive after 7 days. In the middle part of the protocol, data on the age, sex, weight, and the cause of death were recorded for every live-born piglet that died in the first week after birth. Additionally, for crushed piglets, diseases, malformations, and the information whether or not the piglet was cross-fostered, had to be recorded. Finally, in free-form text boxes in the lower part of the protocol, the farmers were asked to fill in information on health problems and medical treatments applied to the sow and her litter.

All farmers were provided with the same model of weighing scale (Küchenwaage elektro Prima Vista, Landi Schweiz AG, Dotzigen, Switzerland) to measure the weight of dead piglets. To record the ambient temperature, they were given a temperature logger (UA-001-64 Hobo Pendant 64 K Temp-Alarm Data Logger, Onset Computer Corporation, Bourne, Massachusetts) for each farrowing room (1 to 6 per farm). They were instructed to place the temperature logger in the middle of the room, at head height of the sow (~1 m above ground) and out of reach for the animals. The temperature was logged at a frequency of 1 h^−1^ and data were retrieved by the authors.

### 2.3. Recorded events

Based on the farmers’ records, piglet losses in the first week after birth were assigned to five categorical events. As it was not possible to compare the producer-recorded causes of PWM with post-mortem diagnoses, we followed recommendation by Vaillancourt et al. (23) and defined events that allow little interpretation (see list below). The first event, *piglet loss*, represented all piglet losses of live-born piglets in the first week after birth. Following the example of Weber et al. (13, 28), we further differentiated the event *piglet loss* into the events *not crushed* and *crushed* based on the farmers’ judgement of the cause of death. Vaillancourt et al. (23) reported that farmers consistently were able to identify piglets that had been crushed, but frequently misidentified piglets dying from other causes than crushing. Finally, we differentiated the event *crushed* into the events *low viable crushed* and *viable crushed* based on body weight and signs of weakness, as recorded by the farmers. Christensen and Svensmark (35) observed that the sensitivities of the mortality categories were higher when the clinical signs recorded by the farmers were included in the diagnosis.

Addition to event *low viable crushed*: Poor health state due to diseases, e.g., diarrhea, or malformations, e.g., splay legs, as recorded by farmers, was considered as sign of weakness. Piglets with a body weight of less than 1 kg were defined as absolutely underweight. This is a common rule for breeding purposes in Switzerland (38). Our definition of absolute underweight included underweight piglets at birth (38, 39) and absolutely underweight piglets during the whole study period (first week after birth). Dead piglets were defined as relatively underweight, if their weight was less than the sum of a minimum normal birth weight of 1 kg plus an average daily weight gain of 200 g. Therefore, relatively underweight piglets either were born absolutely underweight or had an average daily weight gain less than 200 g, or both. These 200 g of average daily weight gain for healthy piglets before weaning are based on literature (39, 40) and on personal experience of the first author in a previous study in free farrowing pens with piglets in their first 5 days after birth (unpublished data).

Definitions of the events:

- *Piglet loss* = A live-born piglet died in the first week of life.
- *Not crushed* = A live-born piglet died in the first week of life and was judged by the farmer not to be crushed by the sow (died spontaneously or was appropriately killed by farmer).
- *Crushed* = A live-born piglet died in the first week of life and was judged by the farmer to be crushed by the sow.
- *Low viable crushed* = A live-born piglet was judged by the farmer to be crushed by the sow while being absolutely or relatively underweight and/or having signs of weakness (= secondary crushing loss).
- *Viable crushed* = A live-born piglet was judged by the farmer to be crushed by the sow without being underweight and/or having signs of weakness (= primary crushing loss).

### 2.4. Statistical analysis

Finally, in the statistical analysis we considered 9,543 live-born piglets out of 740 litters with complete data records with respect to characteristics of the dead piglets (birth state [live-born vs. stillborn], death date, and body weight of crushed piglets), the litter (number of live-born piglets, number of stillborn piglets, number of total piglet losses, and information about cross-fostering), the sow (parity class, gestation length, and farrowing date), and the environment (temperature in farrowing room). In total, 123 litters were excluded from statistical analysis as records were incomplete.

We performed mixed-effects Cox regression survival analysis using R (version 4.2.2; R Core Team 2022) and the R package coxme (41). Separate regression models were fitted to analyze the time to occurrence of one of the five events. Piglets that survived the 7-day study period or died on days 0–7 from a different event than the specific one defined for the respective model were censored, as is appropriate for Cox regression. The random and fixed effect structure was identical across all models. Litter identifier nested in farm identifier were set as random intercepts. The parity class, gestation length, number of live-born piglets, number of stillborn piglets, and the ambient temperature in the farrowing room on the day before death were included as fixed effects. The temperature was calculated as the number of hours with a temperature above 30°C. The approach of aggregating the hourly data to daily temperature data was selected from a large set of candidate methods. Candidate hourly-to-daily temperature aggregation methods included hours with temperature above a certain value (21–32°C), mean temperature above the upper boundary [mean (max (0, *T*°C–22°C))] of the optimal temperature range (18–22°C) as recommended for farrowing rooms in Western Europe (36, 42), as well as a large set of statistical measures for central tendency, variability, and distribution. From these candidates, daily hours with *T* > 30°C was selected, because, when temperature was aggregated in this way and used as fixed effect, this resulted in the best model for the response variable representing time to the event *crushed*. Interestingly, 30°C is the minimum temperature prescribed in Switzerland for the piglet creep area in the first days after birth (43) and Weber et al. (13) assumed a higher crushing risk when the room temperature increases toward the nest temperature. Cross-fostering was conducted at unknown time points in relation to birth. Consequently, it was not possible to consider cross-fostering in the survival analysis.

## 3. Results

### 3.1. Descriptive data analysis

**Figure 1:**
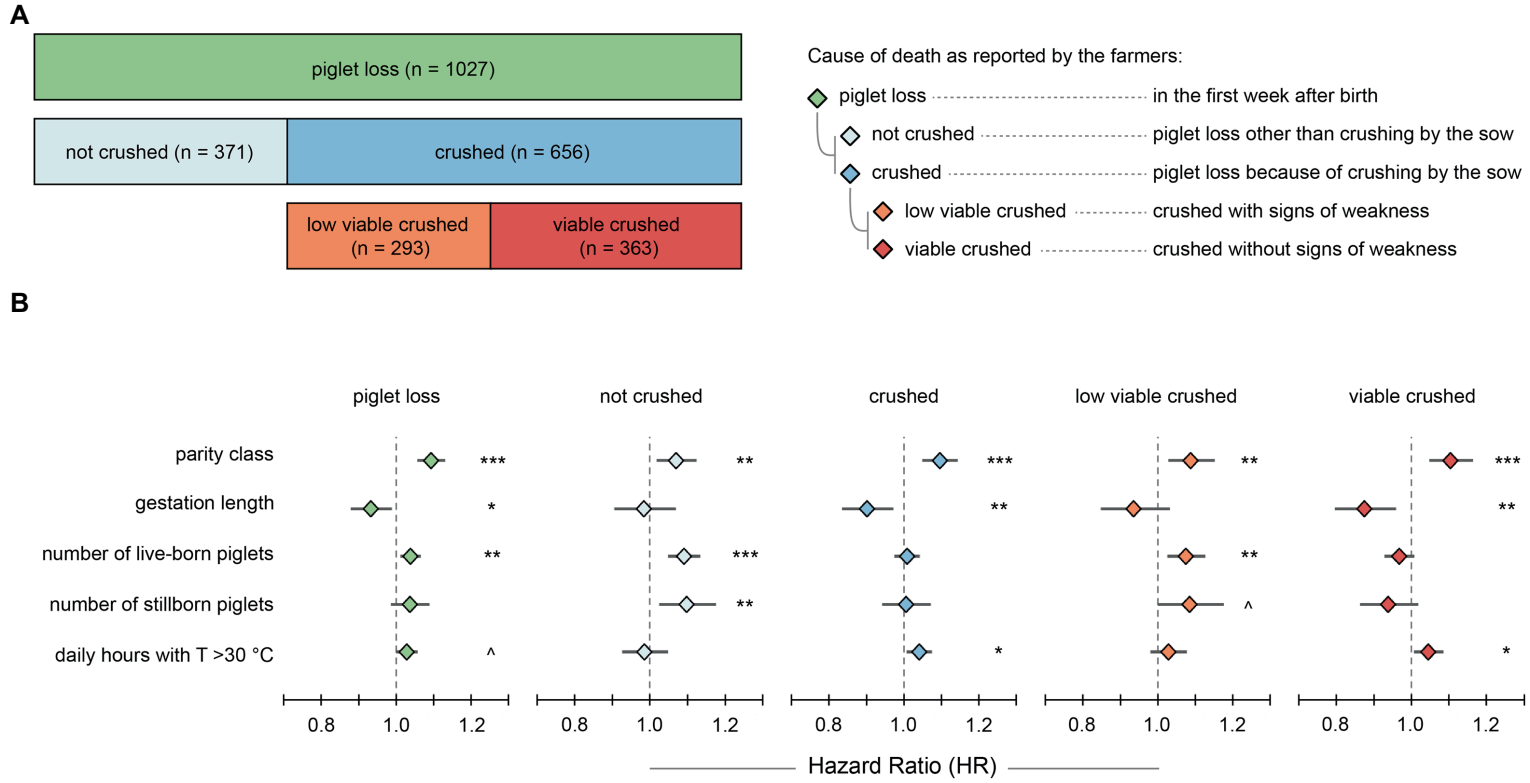
**(A)** Differentiation of the events for the number of piglets that died in the first week after birth. The bar on top represents all *piglet losses*, the bars in the middle the differentiation of the *piglet losses* into *not crushed* versus *crushed* piglets, and the bars at the bottom the differentiation of the *crushed* piglets into *low viable crushed* versus *viable crushed* piglets. **(B)** Results of the mixed-effects Cox regression analyses for the five defined events. Estimated hazard ratios with 95% confidence interval are shown for the five co-variates (potential risk factors). Significance Code: ***p < 0.001, **p < 0.01, *p < 0.05, ^*p* < 0.1.

Table 1 provides information on the number of litters, sow characteristics, and litter performance per farm. In total, 10,567 piglets were born in the 740 litters of the data set, corresponding to 14.3 piglets born per litter on average. Thereof, 1,024 piglets were recorded as stillborn, resulting in an average stillborn rate of 9.7% and an average of 12.9 live-born piglets per litter. Average gestation length was 116.6 days. In total, 1,027 of 9,543 live-born piglets (10.76%) died in the first week after birth. These are henceforth referred to as *piglet losses* and were assigned to the above defined events as follows: 371 (36.1%) *not crushed*; 656 (63.9%) *crushed*, of which 293 (44.7%) were *low viable crushed* and 363 (55.3%) were *viable crushed* (Figure 1A). Cross-fostering was carried out in 406 of 740 litters (54.9%), and only 12 out of 656 *crushed* piglets (1.8%) had a history of cross-fostering.

**Table 1:** Information on number of litters included in the analysis, sow characteristics, and litter performance per farm.

Farm	Total number of recorded litters	Average parity class	Average gestation length	Average total number of piglets born per litter	Average number of live-born piglets per litter	Average stillborn rate (%)
1	63	4.5	116.6	15.7	14.2	9.6
2	101	4.2	116.2	14.0	12.8	8.7
3	35	4.6	116.9	15.0	13.2	12.2
4	58	4.9	116.3	13.4	12.6	5.9
5	150	3.2	116.3	14.3	12.8	10.8
6	18	3.6	116.9	16.0	14.9	6.6
7	112	4.0	117.2	14.3	13.0	9.2
8	72	4.0	115.5	13.9	12.8	8.1
9	69	4.3	117.0	13.4	11.9	11.3
10	27	4.6	117.6	14.1	11.9	16.0
11	35	3.5	117.1	14.3	13.0	8.6

### 3.2. Survival analysis

We used individual mixed-effects Cox regressions to statistically model the instantaneous hazard (probability) of dying at any given time in the first week after birth by one of the five defined events (*piglet loss*, *not crushed*, *crushed*, *low viable crushed*, and *viable crushed*). Figure 1B shows the estimated hazard ratios (HRs) for the co-variates (parity class, gestation length, number of live-born piglets, number of stillborn piglets, and daily number of hours with a temperature of >30°C). The HR represents the factor by which an unknown baseline hazard multiplies when the co-variate of interest increases by one unit, i.e., HRs of 1.1 and 0.9 correspond to a 10% increase and a 10% decrease in hazard, respectively, per unit increase of the co-variate.

#### 3.2.1. Parity class

With every additional parity of the sow the hazard for a piglet to die at any given time in the first week after birth (*piglet loss*) increased by 9.3% (HR [95% confidence interval]: 1.09 [1.06, 1.13]). Irrespective of whether death was caused by crushing (*crushed*) or other causes (*not crushed*), a higher parity class was associated with increased hazard. The hazard for *not crushed* increased by 7.0% (HR: 1.07 [1.02, 1.12]) whereas that for *crushed* increased by 9.6% (HR: 1.10 [1.05, 1.14]). Moreover, irrespective of the presence (*low viable crushed*) or absence (*viable crushed*) of weakness in a crushed piglet, a higher parity class was associated with increased hazard. For *low viable crushed* the hazard increased by 8.8% (HR: 1.09 [1.03, 1.15]) and for *viable crushed* it increased by 10.4% (HR: 1.10 [1.05, 1.16]).

#### 3.2.2. Gestation length

With every additional day of gestation (increasing gestation length) the hazard for a piglet to die at any given time in the first week after birth (*piglet loss*) decreased by 6.8% (HR: 0.93 [0.88, 0.99]). Similarly, a decrease in hazard was apparent for *crushed* (9.9%; HR: 0.90 [0.84, 0.97]) and for being crushed without signs of weakness (*viable crushed*, 12.6%; HR: 0.87 [0.80, 0.96]). No support for an effect of gestation length was found for dying by other causes than crushing (*not crushed*; HR: 0.98 [0.91, 1.07]) and for *low viable crushed* (HR: 0.93 [0.85, 1.03]).

#### 3.2.3. Number of live-born piglets

With every additional live-born piglet in the litter the hazard for a piglet to die at any given time in the first week after birth (*piglet loss*) increased by 3.8% (HR: 1.04 [1.01, 1.07]). Similarly, an increase in hazard was apparent for *not crushed* (9.0%; HR: 1.09 [1.05, 1.13]) and for being crushed with signs of weakness (*low viable crushed*, 7.5%; HR: 1.08 [1.03, 1.13]). No support for an effect of the number of live-born piglets on the hazards for *crushed* (HR: 1.01 [0.97, 1.04]) and *viable crushed* (HR: 0.97 [0.93, 1.01]) was found.

#### 3.2.4. Number of stillborn piglets

With every additional stillborn piglet in the litter the hazard for a piglet to die by other causes than crushing at any given time in the first week after birth (*not crushed*) increased by 9.8% (HR: 1.10 [1.02, 1.18]). For *low viable crushed* a statistically weakly supported effect was found, the hazard increased by 8.4% (HR: 1.08 [1.00, 1.18]) with every additional stillborn littermate. No support was found for effects on hazards for *piglet loss* (HR: 1.04 [0.99, 1.09]), *crushed* (HR: 1.01 [0.94, 1.07]), and *viable crushed* (HR: 0.94, [1.01, 1.09]).

#### 3.2.5. Daily number of hours with a temperature of >30°C

With every additional hour with an ambient temperature above 30°C the hazard for a piglet to die by crushing at any given time in the first week after birth (*crushed*) increased by 4.0% (HR: 1.04 [1.01, 1.07]) and that for *viable crushed* by 4.5% (HR: 1.05 [1.01, 1.09]). A statistically weakly supported effect was found for *piglet loss*, the hazard increased by 2.8% (HR: 1.03 [1.00, 1.06]). No support was found for an effect of the temperature on the hazards for *not crushed* (HR: 0.99 [0.93, 1.05]) and *low viable crushed* (HR: 1.03 [0.98, 1.08]).

## 4. Discussion

### 4.1. Survival analysis

#### 4.1.1. Parity class

In the present study, a higher parity class was associated with an increased hazard for the piglets to die at any given time in the first week after birth, irrespective of whether death was caused by crushing or by other reasons (*not crushed*). The association between parity class and general PWM in primiparous versus in multiparous sows is well studied (e.g., 12, 26, 44, 45). A lower colostrum yield and quality in primiparous compared with multiparous sows makes piglets of first parity sows more prone to diseases (2, 46–48). However, in our study we found a higher PWM with increasing parity of the sow. This finding might be explained by three main factors. First, in general, older sows have a longer farrowing duration (49), which increases the probability of intrapartum hypoxia and subsequently reduces neonatal viability (2). Second, the variability of the litter size and birth weight increases with increasing parity class leading to a higher probability of more underweight piglets (2, 3, 50). Third, older sows usually have reduced function and accessibility of teats (2, 45, 51), increasing the inequality in feeding among the piglets (45).

For piglet mortality due to crushing, the conclusions of available studies on potential effects of parity class are inconsistent. In contrast to our results, Vrbanac et al. (5) and Pandolfi et al. (25) found a lower crushing risk for later parities when compared to the first and second parity. Jeon et al. (52) and Ostović et al. (53) reported no association between parity class and crushing risk. Consistent with our results, several other studies reported that a higher parity class was associated with an increased crushing risk (e.g., 3, 8, 26, 29, 54). Vieuille et al. (55) described a higher reactivity of piglets in litters of first parity sows, they seemed quicker to move away from the mother when she suddenly changed her position. Olsson et al. (3) gave two additional explanations for higher crushing losses with higher parity class. First, maternal responsiveness might decrease with increased parity class owing to older sows being heavier and clumsier and having more health problems, e.g., claw or leg problems and teat damage (3). Second, older sows have larger litters and more underweight piglets (3). Koketsu et al. (26) combined in their analyses crushed piglets which had died because of trauma and those characterised by low viability and found that, in parity 3 to 5 and more, piglets had higher mortality ratios than piglets from sows of parity 1 or 2. In line with this reasoning, we expected that the weaker piglets would be crushed in litters of older sows, which is supported by our results.

#### 4.1.2. Gestation length

We found statistical support that with increasing gestation length the hazard to die at any given time in the first week (*piglet loss*) and to die *crushed* and *viable crushed* decreased. Such a decrease in general PWM with longer gestation was shown in the investigations of Hofer (56), based on Swiss genetics with an average number of <12.6 live-born piglets per litter (28, 57), and Hales et al. (30), based on an average number of 15.5 live-born piglets per litter. Hales et al. (30) showed that piglets born before day 116 of gestation had an increased risk of dying compared with piglets that were born later. Hanenberg et al. (58) and Rydhmer et al. (59) hypothesized that selection for longer gestation would probably improve piglet survival. Rydhmer et al. (59) showed a high heritability of the gestation length and positive genetic correlations between gestation length and average birth weight. Vice versa, the selection for piglet survival results in a longer gestation length. In Switzerland, breeding goals changed in 2004, when the breeding value ‘piglet survival rate’ was introduced (60). Since then, it is given the highest importance in the damlines (61, 62), whereas increasing the litter size is no longer a breeding focus (61). Hofer (56) observed a continuous increase in gestation length until 2014 and hypothesized that this high importance of the ‘piglet survival rate’ resulted in an increase of the gestation length, leading to more mature piglets even in larger litters and finally a decrease in PWM. Furthermore, Rydhmer et al. (59) found positive genetic correlations between gestation length and piglet growth rate during the first 3 weeks. Therefore, it is likely, that maturation and growth rate not only influence general PWM, but crushing risk in particular. Vallet and Miles (63) hypothesized that the impairment of coordination and reflexes due to reduced brain myelination could decrease the ability of small piglets to avoid the sow when necessary, and, therefore, may contribute to the risk of crushing. This hypothesis is supported by the findings of Amdi et al. (64), who found a tendency of higher vitality score in normal (normal birth weight and head morphology) piglets compared to piglets with severe intrauterine growth-restriction (IUGR). IUGR piglets have a higher risk of dying in the first days after birth (30, 65), when crushing risk is highest.

#### 4.1.3. Number of live-born piglets

We found that the general hazard to die at any given time in the first week (*piglet loss*) and to die by a weakness-associated event (*not crushed* and *low viable crushed*) increased with increasing number of live-born piglets in the litter. No support was found for an effect of the number of live-born piglets on the hazards for *crushed* and *viable crushed*.

Several studies reported higher PWM associated with larger numbers of live-born piglets in the litter (e.g., 3, 12, 13, 26, 28, 66). An association between litter size and weakness-associated deaths was expected because a large litter size, which corresponds generally to a large number of live-born piglets, is strongly associated with a larger number of underweight (3, 50) and IUGR piglets (20, 65). Moreover, in litters with more live-born piglets, each piglet gets less colostrum, as colostrum yield is reported to be independent of litter size (67). Particularly in piglets with a low birth weight, a reduced colostrum intake leads to weakness and consequently a higher PWM risk (39, 47, 68).

As reviewed by Ward et al. (27) litter size was identified as a contributing factor towards higher crushing incidence across pig breeds (29, 69). Liu et al. (70) hypothesized that larger litter size may cause crowding and leave piglets less space to withdraw while sows are lying down or getting up, which increases the risk of crushing. Additionally, higher crushing losses in larger litters can be explained by the fact that there is more fighting for access to the teats leading to disturbance of the suckling process, more position changes of the sow, and, therefore, a higher risk for crushing (61). In contrast to these results, we did not find statistical support for an effect of the number of live-born piglets on general crushing risk and on crushing risk of viable piglets. Analyzing a large dataset from Swiss commercial farms, Weber et al. (28) reported that with a larger litter size at birth, significantly more losses occurred due to all reasons (total, crushed, others), but while the number of losses other than crushing increased strongly, crushing losses increased only slightly. An explanation might be related to relatively small average litter sizes in Switzerland and to cross-fostering management. To handle larger litters, cross-fostering of heaviest piglets (71, 72) between litters is a very important method to equalize litter size, with the aim to secure milk to the piglets (71). Thus, piglets in equalized large litters tend to have better survival chances (72, 73).

#### 4.1.4. Number of stillborn piglets

With every additional stillborn piglet in the litter the hazard for a piglet to die in the first week after birth by other causes than crushing (*not crushed*) increased. Additionally, weak statistical support for such an increase was also found for *low viable crushed*. But we found no support for an effect of the number of stillborn piglets on the hazard for the event *piglet loss*, which is in concordance with the finding of Koketsu et al. (26).

Depending on the time of infection, a combination of stillborn and low viable piglets at birth can be caused by porcine reproductive and respiratory syndrome virus (PRRSV), Aujeszky’s disease virus (ADV), classical swine fever virus (CSFV), porcine parvovirus (PPV), porcine circovirus 2 (PCV-2), and leptospira (74). At the time of this study, Switzerland was approved to be free from PRRSV, ADV, and CSFV (75, 76) and just a single case of leptospirosis in pigs was reported in a distance of at minimum 100 km of all study farms (77). Moreover, all farms in this study vaccinated the sows against PPV and cases of PCV-2 induced reproductive failures were described to be relatively rare in Switzerland (78). Therefore, the observed effects of the number of stillborn piglets in the litter on the hazard to die from a cause of death related to weakness (*not crushed*, *low viable crushed*) can likely be explained by non-infectious rather than infectious causes. As reviewed by Muns et al. (2), intrapartum hypoxia suffered by piglets at birth is one of the most important causes of stillbirth and early PWM in piglets and directly related to neonatal viability. A reduction in the oxygenation of prenatal piglets, compromising their viability, can be caused by uterine contractions in sows with a long farrowing duration (2). Factors leading to a longer farrowing duration, i.e., high parity, large litters, and low back fat levels in sows, are associated with a higher stillborn rate (79). Because a prolonged farrowing duration results in an elevated number of weak or stillborn piglets, sows are often treated with oxytocin, which decreases the duration of farrowing (80, 81) but also increases the number of stillborn piglets (81). The routine administration of oxytocin immediately after the birth of the first piglet or overdosing of oxytocin can compromise piglet viability [reviewed by Muns et al. (2)] and might explain our results besides long farrowing durations. Unfortunately, we can only speculate about the use of oxytocin in our study, as this data is not available in our records.

#### 4.1.5. Temperature

With every additional hour with an ambient temperature above 30°C the hazard for *crushed* and *viable crushed* increased. Our results are in line with the observations made by Weber et al. (12, 13) and what many farmers report; crushing losses especially of viable piglets are generally more frequent in summer than in the other seasons. As mandatory according to the Swiss Animal Protection Ordinance (33, 43), every farm included in this study had a heated piglet creep area integrated into the farrowing pen, to satisfy the completely different thermal demands of the sow and the piglets. In the first 3 days after birth, a minimum temperature of 30°C is prescribed in the piglet creep area independently of the season (43). As shown in several studies (82–84), the acceptance of the heated creep area by the piglets is low in the first days after birth, when the crushing risk is highest (82). Even lower is the acceptance by the piglets when the temperature difference between sow area and piglet creep area is small (83, 85, 86). Viable piglets spend less time in the nest away from the sow’s body when the room temperature increases toward the nest temperature (13, 83), which would elevate the risk of being crushed, as assumed by Weber et al. (13). This was confirmed by Gao et al. (86), who found a crushing mortality rate of 15.2% in a room with an air temperature of 30.4°C and the same temperature in the piglet creep, while in a colder room with 15.3°C and a piglet creep temperature of 25.9°C no piglet was crushed [reviewed by Liu et al. (70)]. Furthermore, Jeon et al. (52) found a higher crushing rate in summer than in other seasons, which they attributed to greater heat stress experienced by the sows. Heat stress can cause alterations in sow behavior, such as a higher activity leading to a reduction of the piglets in the amount and duration of suckling, which might in turn be related with higher piglet mortality due to crushing (87). However, the air temperature has to be relatively high (above 27°C) before it affects feed intake, milk yield or weight loss of the sow, and consequently the daily weight gain of litters, as reviewed by Bjerg et al. (88).

### 4.2. Summarizing crushing risk for viable versus low viable piglets

We hypothesized that there are differences in risk factors between the events labelled as *viable crushed*, i.e., being crushed in viable state, and *low viable crushed*, i.e., being crushed in low viable state. Our results support this hypothesis, as we identified two risk factors for *viable crushed* that were not identified as risk factors for *low viable crushed*. These were shorter gestation length and higher ambient temperature. Vice-versa we identified two risk factors for *low viable crushed* that were not identified as risk factors for *viable crushed*, namely higher number of live-born piglets and higher number of stillborn piglets (the latter with only weak statistical support). Additionally, we expected that risk factors for dying from other causes than crushing (*not crushed*), typically related to weakness, would be more similar to those for *low viable crushed* than to those for *viable crushed*. This is supported by our results as the risk factors identified for *not crushed* were the same as those identified for *low viable crushed* (number of stillborn piglets and number of live-born piglets) but differed to the risk factors identified for *viable crushed*.

## 5. Conclusion

This study shows the importance of a differentiation between low viable crushed and viable crushed piglets. A differentiation based on the piglet’s body weight and external signs of weakness (e.g., diseases, malformations) can considerably affect the interpretation of risk factors. We conclude that low viable crushed and viable crushed piglets should be handled as two different causes of death, particularly for breeding and research purposes. Recording underweight or weak piglets simply as ‘crushed’ should be avoided. The results of previous studies not differentiating between low viable and viable crushing losses should be interpreted cautiously. Future studies should differentiate between primary and secondary crushing losses and focus on identifying the risk factors for crushing of viable piglets, because viable piglets are the focus of welfare and economic interests.

## Data availability statement

The raw data supporting the conclusions of this article will be made available by the authors, without undue reservation.

## Ethics statement

The animal study was reviewed and approved by Swiss Cantonal Veterinary Office Thurgau, Frauenfeld, Switzerland. Written informed consent was obtained from the owners for the participation of their animals in this study.

## Author contributions

CS-V was responsible for study design and farm acquisition, organised data collection by farmers, digitalised the farmers’ records, prepared data for statistical analysis, and drafted all other parts of the manuscript. MSi conducted the statistical analysis, visualised data, and drafted the statistical part of the manuscript. MSc critically reviewed the draft and gave substantial input and constructive criticism on the content of the manuscript. BW edited the manuscript. All authors contributed to the article and approved the submitted version.

## Funding

This study was part of a PhD project funded by the Swiss Federal Food Safety and Veterinary Office (grant no. 2.17.04). Open access funding by Agroscope.

## Conflict of interest

The authors declare that the research was conducted in the absence of any commercial or financial relationships that could be construed as a potential conflict of interest.

## Publisher’s note

All claims expressed in this article are solely those of the authors and do not necessarily represent those of their affiliated organizations, or those of the publisher, the editors and the reviewers. Any product that may be evaluated in this article, or claim that may be made by its manufacturer, is not guaranteed or endorsed by the publisher.
